# Supplementary material for: Thirteen dubious ways to detect conserved structural RNAs
Source: IUBMB Life. Author manuscript; Available in PMC 2024 Jul 10. (PMC11234323; doi:10.1002/iub.2694)
Supplement: supplementary_material [file NIHMS1907110-supplement-supplementary_material.gz › supplemental_material/rscape_v2.0.0.k.tar.gz!/rscape_v2.0.0.k/lib/R2R/R2R-current/NotByZasha/nlopt-2.6.2/src/algs/stogo/techreport.pdf]

A C++ Programme for Global Optimization

Serguei Zertchaninov*and Kaj Madsen

Abstract A stochastic Branch-and-Bound Method for global optimization over a compact right parallelepiped, parallel to the coordinate axes, has been developed, described and tested in ([1],[2]).
This report describes the C++ implementation of the method. Section 2 is a short description of the new method, Section 3 provides some implementation details, and Section 4 is a user's guide to the programme.
The source code of the implementation is available on request to the authors.

1 Introduction

It is well known that many problems in industry and economy can be formulated as mathematical programming problems, i.e. as the minimization of a function $f : D \to \Re$ where $D \subseteq \Re^n$. Dimensionality n of such functions usually is quite large. Besides that, very often the function f has several local minima in the region of interest. However, only the smallest one is interesting:

$$f^* \equiv inf\{f(x) \mid x \in D\} \tag{1}$$

f^* is called the *global minimum* and the set of points where it is attained is called the set of *global minimizers.*

The problem of creating an algorithm (a computer program) which is guaranteed to find this set has not been solved, and it probably will never be. Interval analytic methods only solve the problem for a limited class of problems. Most other methods either give no guarantee for convergence or convergence is so slow that for larger dimensions it is unrealistic. Some techniques require 'help' of a skilled engineer who uses his insight into the technical problem which is modeled. This can lead to solutions which could not have been found by fully automatic optimization methods.

The purpose of this project is to develop an automatic optimization method. Having the exponential complexity of the global optimization problem in mind we don't intend to guarantee the solution is found, but rather to try to get the best possible out of a given amount of computational resources. The user should specify how much computer time and storage he will spend rather than specifying some epsilon accuracy as required by many traditional approaches.

Since the classical deterministic method based on interval arithmetic has been very successful we base our new technique on the same general branch-and-bound principle,

*on leave from Informatics Department, Nizhnij Novgorod State University

but without the requirement that interval arithmetic must be applicable. Thus the new method is a strategy for managing local methods (like quasi-Newton methods) in a branch-and-bound search for global minimizers.

This report consists of several sections. In Section 2 we give a general description of the new method. The details of implementation are given in Section 3. Recommendations in Section 4 tell how to use this program. In Section **??** we provide some computational results.

2 Description of the method

We start the description with introducing some terms which will be used in further material. Let *function* be a given real function of n variables. Let *box* be an n-dimensional real interval. We assume that function is defined in some box, i.e. has interval constraints. Let C, or candidate set, be a set of boxes to test. Let G, or garbage set, be a set of boxes temporarily eliminated from the search. We define the value $fbound$ which is the smallest function value found during the search. Temporary value B is used to store a single box. Thus the structure of the new algorithm is the following:

the algorithm: (2)

initialize $\to C, fbound, G$
while true **do**
  while $C \neq \emptyset$ **do**
    remove-best$(C) \to B$
    reduce-or-subdivide$(B) \to result, fbound, garbage$
    $C = C \cup \{result\}$
    $G = G \cup \{garbage\}$
  end
  while $G \neq \emptyset$ **do**
    remove$(G) \to B$
    split$(B) \to result$
    $C = C \cup \{result\}$
  end
end

The *initialize* procedure is called when the method is started the first time, and every time when the method runs out of memory. It clears both C and G sets, then adds the initial box together with the best points of minimum, found in the previous iterations, to C set. Obviously, there are no such points when the method starts the first time. In this case fbound value is also initialized.

The purpose of the outer **while**-loop is to let the program run for as long as the user wants. When time is out the program stops providing the best estimate of the global optimizers that has been found. The longer time used, the better result should be expected. For small dimensions, however, the whole set of global minimizers should normally be found in a rather modest amount of computer time.

Inside the first inner **while**-loop the actual search procedures are being performed. **remove-best** procedure removes the best box from the candidate set and stores it in B. **reduce-or-subdivide** performs two consequential actions. At first, using different testing procedures such as monotonicity test and quasi-newton search, it determines whether box should be eliminated from the candidate set or not. At second, using the conclusion obtained on the first stage, it places the box to the garbage or splits it into two parts for further exploration. Let us take a closer look at how **reduce-or-subdivide** works.

Sampling: To prepare the box for testing method samples a certain amount of points inside the box. It uses two strategies for placing these points. The first way is to use some regular distribution. Method places one point in the center of the box, and for each coordinate it places two points, shifted from the center along the axes to the left and to the right on the same value in percent to appropriate side length. Such point we will call *regular*. The second way is to use random points, uniformly distributed in the box. These we will call *random* points. For each point the function value is calculated.

Monotonicity test: This test uses a certain number of sampled points to check the monotonicity. If it proves function in the box is monotonous the box is placed to garbage. But here we should mention that the monotonicity test is skipped in our implementation because this has proved most efficient in our tests. The possible reason for inefficiency of monotonicity test is the following: after the first loop each full rank box in C contains at least one local minimum (because of *subdivide* below), and thus f is not likely to be monotone in any of these boxes.

Newton-test: From the given number of sampled points method starts local search sequences to find stationary points located inside the box. The local technique used in our tests is a version of the dog-leg method, [3], which allows for an easy control of the step lengths. Gradient in every point generated by local method is used to update *maximal gradient* value. We distinguish three pictures of convergency: If all iteration sequences go out of B then B is placed to garbage. If all starting points lead to iteration sequences converging to the same point $x \in B$ then $\{x\}$ is placed to result and B is added to garbage. If none of the two situations take place then we have found several local minima in B. Then method estimates the lower bound of the function in the box *LB(B)*. It takes all points, generated by sampling procedure, and maximal gradient value to calculate *LB(B)* as follows:

$$LB(B) = \min_{i \neq j} \frac{f(x_i) + f(x_j) - maxgrad\|x_i - x_j\|}{2} \tag{3}$$

LB(B) is corrected if it is greater than the lowest minimum found in the box. Finally, if $LB(B) > fbound$ then box is added to garbage. Else *subdivide* procedure should be performed.

Subdivide: In this case B contains several known local minima, and the splitting into two is done so each new box contains (known) local minima. There are several ways of achieving such a subdivision. We will give more detailed description in the next section.

The first **while**-loop runs until the candidate set is empty. It also can be interrupted if method runs out of memory. In this case the initialization procedure is called. If method runs out of time limit, it stops. If there was no interruption method proceeds with the second inner **while**-loop.

In the second inner **while**-loop the garbage set is emptied. **remove** procedure takes one box from the garbage set and stores it in B. Then this box is split into two parts by **split** procedure. It uses different splitting approaches depending on the amount of stationary points found in the box.

If there are no such points, the box is subdivided in two equal parts by plane, crossing the center of the box and perpendicular to the side with the maximal length. If there is only one stationary point in the box then method finds the coordinate for which the distance from the point to the border is maximal. The splitting plane is chosen to be perpendicular to this coordinate axis, and it is shifted from the point to a certain value in the direction where distance to the border is maximal. In case of multiple stationary points **subdivide** procedure from above is used.

The second **while**-loop runs until the garbage set is empty. It also can be interrupted if method runs out of memory. In this case the initialization procedure is called. If method runs out of time limit, it stops. If there was no interruption method proceeds with the first inner **while**-loop, and so on.

3 Implementation

To implement this method C++ programming language was used. We organized all data and procedures as C++ classes, each representing a quite independent part of the search process. There are two groups of classes: main and supplementary. The main group classes are the following: *TProblem* - a class containing data and routines for calculating function and first derivative values, and for managing the results output; *TMethod* - a class providing procedures for box testing; *Process* - a class which contains all data structures and procedures necessary for managing the search, including TProblem and TMethod objects. The supplementary group contains storage classes: *TTrial* - a class to represent a single point; *TBox* - a class representing a box with points inside; *TBoxList* - a class designed to store ordered list of boxes. There are also two classes to implement vectors and matrices - *TVector* and *TMatrix*, which will not be described here.

Let us consider a supplementary group of classes. We will not describe all data members and functions but only the most important ones. First class is TTrial - quite trivial one as all data are public:

```
class TTrial
{
  public:
    TVector XVal; - vector of real values, or a point
    double FVal; - corresponding function value
    PTTrial Next; - pointer to next TTrial in a list

    TTrial(int); - constructor by size of XVal vector
    TTrial(RTTrial); - copy constructor
    RTTrial operator=(RTTrial); - assignment operator
    int GetXDim(); - returns dimension of XVal vector
    long int SizeOfTrial(); - returns memory required for storing TTrial
```

};

Function SizeOfTrial() returns amount of memory in *sizeof(char)* units required to store one object of TTrial class. It is used in procedures responsible for memory checks. All other functions and data members of TTrial are quite obvious. Class TBox is the second basic storage class, and it looks like following:

class TBox
{
  public:
    TVector Right; - vector of right boundaries
    TVector Left; - vector of left boundaries
    PTTrial Pts; - list of points located in the box
    double CMin; - current minimum in the box
    PTBox Next; - pointer to next TBox in a list

    TBox(int); - constructor by dimensionality
    TBox(RTBox); - copy constructor
     TBox(); - destructor

    int Insert(PTTrial); - inserts trial to the box
    int AddCopy(PTTrial); - inserts copy of trial to the box
    PTTrial GetBest(); - returns pointer to the best trial
    PTTrial RemoveBest(); - removes the best trial from the list
    void Clear(); - clears trials list
    int GetAmount(); - returns number of trials in the list

    int InBox(PTTrial); - returns TRUE if trial is inside the box
    int GetXDim(); - returns dimension of the box
    int GetType(); - returns type of the box
    int NearToMin(PTTrial,double); - checks distance to the points
    void Truncate(double); - discards trials with too high function values
    double GetLongSide(); - returns length of the longest side
    double GetShortSide(); - returns length of the shortest side
    double GetMaxSlope(); - estimates maximal slope of function
    double GetLBound(double); - estimates lower bound of function
    long int SizeOfBox(); - returns memory required for storing TBox
    static long int GetMemUsed(); - returns memory used for trials
};

There is a group of functions performing operations with the list of points. It is possible to insert a point or a copy of the point into the list, obtain pointer to the best point in the list, remove it from the list, get total number of points in the list, and clear the list of points.

Function InBox(PTTrial **tr**) checks whether given point **tr** located inside the box or not. Do not be confused - it does not check whether the point is in the list. Function

GetType() returns one of the predefined box type constants. There are three of them: for empty box, for box containing only one point in its list, and for box with several points inside. Function NearToMin(PTTrial **tr**,double **dist**) checks whether given point **tr** located close to one of the points in the list or not. Parameter **dist** gives the distance limit. Function Truncate(double **band**) removes points with function values greater than CMin + **band**. Function GetMaxSlope() estimates maximal slope of the function in the box using points from the list. The maximal slope is calculated as follows:

$$MS = \max_{i \neq j} \frac{\|f(x_i) - f(x_j)\|}{\|x_i - x_j\|} \tag{4}$$

Function GetLBound(double **slope**) estimates lower bound of the function in the box using point from the list. Is uses Formula 3 for calculations, where *maxgrad* is given by **slope** parameter. Function SizeOfBox() returns amount of memory in *sizeof(char)* units required to store one object of TBox class. Function GetMemUsed() returns amount of memory used by all TTrial objects stored in all TBox objects available. Two last functions are used in procedures responsible for memory checks.

Class TBoxList serves for storing ordered list of boxes. Boxes in the list are ordered by their CMin values in increasing order. So the best box is always at the beginning of the list. There are the same basic operations for including and removing a box as in class TBox for points. Definition of TBoxList looks like following:

```
class TBoxList
{
    PTBox List; - list of boxes
    static long int BCount; - memory counter for stored boxes
  public:
    TBoxList(); - default constructor
     TBoxList(); - destructor

    int Insert(PTBox); - inserts box in the list
    int AddCopy(PTBox); - inserts copy of the box in the list
    PTBox GetBest(); - returns pointer to the best box
    PTBox RemoveBest(); - removes the best box from the list
    void Clear(); - clears list of boxes
    int GetAmount(); - returns number of boxes in the list

    int IsEmpty(); - returns TRUE if list is empty
    static long int GetMemUsed(); - returns memory used for boxes
};
```

Function IsEmpty() returns boolean true in case when list of boxes is empty. Function GetMemUsed() returns amount of memory used by all TBox objects stored in all TBoxList objects available.

Let us consider now the main group of classes. There are three classes in this group, and we start with class TProblem. As it was mentioned above TProblem contains data

and routines for calculating function and first derivative values, and for managing the results output. It is defined like follows:

class TProblem
{
double FBand; - band for minimal function values
double CMin; - current estimation of minimum

long int FCount; - number of function calculations
long int DFCount; - number of derivative calculations

FILE* ResFile; - results file pointer
char* ResName; - results file name

protected:
int OpenResults(); - opens results file
int CloseResults(); - closes results file
int WriteResults(); - writes to results file

virtual void PureCalcF(PTTrial)=0; - calculates F(X)
virtual void PureCalcDF(PTTrial,PTVector)=0; - calculates F'(X)
virtual void PureCalcF_DF(PTTrial,PTVector)=0; - calculates F(X) and F'(X)

public:
TBox Bounds; - area of definition and points of minimum

TProblem(PTBox,double,char*); - constructor by bounds,fband,filename
virtual TProblem(); - destructor

int CalcF(PTTrial); - calculates F(X)
int CalcDF(PTTrial,PTVector); - calculates F'(X)
int CalcF_DF(PTTrial,PTVector); - calculates F(X) and F'(X)
int InBounds(PTTrial tr); - point is in bounds
int AddToResults(PTTrial); - adds trial to results list
int GetXDim(); - returns dimension of the function
double GetCMin(); - returns current minimum
double GetFBand(); - returns function band for minima
long int GetFCount(); - returns number of F(X) calculations
long int GetDFCount(); - returns number of F'(X) calculations
};

Within the definition of the class one can see three pure virtual functions. They were introduced to allow for different implementation of function and first derivatives. User needs to derive classes based on TProblem class where those virtual functions will be defined. Currently we have two such classes ready to use: class *TProblemFF* for calculating function given as a C procedure, and class *TProblemDD* for calculating first derivatives

using simple divided differences. They are incomplete classes because only function or derivative calculations are implemented in each one. But to make a useful class we need to combine these two calculations. So we created class TProblemFFDD derived from both of them. This class is actually used in our program.

Let us return to the class TProblem. Functions PureCalcF(),PureCalcDF(), PureCalcF_DF() are virtual functions calculating function and first derivative values without any border checks and updating any internal variables. These checks and updates are made by corresponding functions without *Pure-* prefix, from which those virtual functions are called. Namely, they check given point for being located within given problem boundaries, update current minimum value CMin, and increment corresponding counters - FCount and DFCount.

Another group of procedures is responsible for creating the results output. *ResFile* and *ResName* are results file pointer and results file name respectively. File is opened by procedure OpenResults(), information is written by WriteResults(), and it is closed by CloseResults().

The result this method provide is a set of stationary points with best function values found during the search. This set of points is stored in the internal list of *Bounds* box. To prevent TProblem from storing points with too high function values *FBand* data member is used. Thus only points with function values less or equal to CMin + FBand will be stored. It is recommended to set FBand to some small value, equal or close enough to the method accuracy, in case if user wants to get only possible global minimizers as a result. Function AddToResults() adds a point to the results list.

The second main class to consider is TMethod - a class providing procedures for box testing. Its definition is following:

```
class TMethod
{
    double Accuracy; - search accuracy
    double AttractR; - radius of attraction
    int RandAmnt; - number of random points
    int NewtAmnt; - number of points to start newton search

    int DogLegStep(PTProblem,PTBox,RTTrial,RTVector,
                   RTMatrix,RTMatrix,double&); - performs dog-leg iteration

  protected:
    void FillRegular(PTProblem,PTBox); - samples regular points
    void FillRandom(PTProblem,PTBox); - samples random points
    virtual void FillBox(PTProblem,PTBox); - samples points in the box
    virtual double DoSearch(PTProblem,PTBox,PTBox); - searches for minima

  public:
    TMethod(double,double,int,int); - constructor by accuracy,attr.R,random,start
    int CheckBox(PTProblem,PTBox); - checks function behavior in the box
    double GetAccuracy(); - returns accuracy
```

double GetAttractR(); - returns radius of attraction
int GetRandAmnt(); - returns number of random points
int GetNewtAmnt(); - returns number of starting points
};

There is only one single procedure which is called from TProcess object to check a box - it is CheckBox(). It corresponds to **reduce-or-subdivide** procedure from Section 2. A box to test is passed together with the problem to this function.

Sampling is made by function FillBox() which, in its turn, calls functions FillRegular() and FillRandom() to sample regular and random points respectively. The number of random points to sample is given by *RandAmnt* data member. We use standard library random generator **rand()** with uniform distribution. FillBox is defined virtual to allow user to implement some different sampling strategy.

Local search is done by function DoSearch() which starts search sequences from the number of sampled points given by *NewtAmnt* data member. It takes only the best *NewtAmnt* points to start. The first box in the parameters list is an original box, and all stationary points, which will be found during the local search, are added to this box. The second box is one with a set of sampled points. DoSearch() returns real value - maximal gradient component found, later used to estimate the lower bound of the function in the box. Data member *Accuracy* gives an accuracy of local search. *AttractR* data member gives the radius of a hypersphere, surrounding each point of local minimum. It is needed to prevent local search from converging to the same point several times. Its value depends significantly on the technique for calculating derivatives, and on the local search method itself. Ideally it should be equal to accuracy, but in our tests we used a greater value. In this implementation we use divided differences for calculating derivatives and dog-leg local search procedure.

The result of applying CheckBox() is one of the predefined constants describing what should be done with the box. There are two possible actions: eliminate box from the candidate set, or split it into two parts placing them back into candidate set. These actions are carried out by TProcess object.

Class TProcess contains all data structures and procedures necessary for managing the search, including TProblem and TMethod objects. It is defined like follows:

class TProcess
{
PTProblem Pr; - pointer to problem object
PTMethod Met; - pointer to method object

long int MaxBytes; - memory limit
long int MaxTime; - time limit
time_t StartTime; - start time

protected:
int SplitBox(PTBox); - splits given box in two

```
public:
   TBoxList Unexplored; - candidate list
   TBoxList Eliminated; - garbage list

   TProcess(PTProblem,PTMethod,long int,long int);
            - constructor by problem,method,memory,time
   int PrepareLists(); - prepares lists of boxes for performing a cycle
   int DoIteration(); - performs one iteration of the method
   int DoProcess(); - performs whole search process
   int InMemory(); - returns memory status
   int InTime(); - returns time status
   long int GetMaxBytes(); - returns memory limit
   long int GetMaxTime(); - returns time limit
   long int GetMemory(); - returns amount of used memory
   long int GetTime(); - returns time from start
};
```

Pointers to TProblem and TMethod objects are passed by constructor parameters and stored afterwards in *Pr* and *Met* data members. Thus they should be created outside the TProcess object. It gives a good flexibility as different problem and method implementations can be used.

There are two objects of TBoxList class in the public area - *Unexplored* and *Eliminated*. They correspond to the candidate and garbage sets respectively. We have placed them both in a public area in order to allow user to get access to the information they contain.

The main procedure which performs the whole search process is DoProcess(). It quits when the time limit given by *MaxTime* data member is finished. Its internal structure is quite simple and looks like that:

```
int DoProcess()
{
  while (PrepareLists())
    while (DoIteration());
  return TRUE;
}
```

The outer **while**-loop here corresponds exactly to the outer loop in the description of the method from Section 2. It runs until the value returned by PrepareLists() function becomes false, which happens when the time limit is finished.

Function PrepareLists() contain both initialization procedure and second outer loop described in Section 2. It initialize the internal data when it is necessary namely in case if process starts the first time, or if it is restarted due to running out of memory. Then, in a **while**-loop, it fetches boxes from the Eliminated list to split them and put into the Unexplored list.

The inner **while**-loop here corresponds exactly to the first inner loop in the description of the method from Section 2. All actions inside the loop are carried out by DoIteration() procedure which is shown below:

```
int DoIteration()
{
  if (!InTime() || !InMemory()) return FALSE;
  PTBox temp=Unexplored.RemoveBest();
  if (temp==NULL) return FALSE;
  if (Met->CheckBox(Pr,temp)==ACTION_SPLIT) SplitBox(temp);
  else Eliminated.Insert(temp);
  return TRUE;
}
```

The first **if** operator checks for time and memory conditions. In the second line the best box from the candidate set is removed and stored in temporary variable. The second **if** operator checks whether candidate set was empty or not, as a nill pointer is returned in a case of empty set. The last **if** operator calls method function to check the box, and a resulting value is compared with one of the predefined action constants. If returned value is equal to ACTION_SPLIT then SplitBox() procedure is applied to the box. Else, namely when returned value is equal to ACTION_ELIMINATE, the box is placed into the garbage set.

Function SplitBox() is called every time a box has to be split, either from inside of PrepareLists() function or DoIteration() function. It always splits a box in two parts and places those into the candidate set. The initial box is deleted but all the points it contained are distributed to new pair of boxes. The procedure recognizes three types of boxes and applies different split subroutines for each type. Empty box is subdivided in two equal parts by plane, crossing the center of the box and perpendicular to the side with the maximal length. If there is only one stationary point in the box then coordinate, for which the distance from the point to the border is maximal, is selected. The splitting plane is chosen to be perpendicular to this coordinate axis, and it is shifted from the point to a certain value in the direction where distance to the border is maximal.

In case of multiple stationary points several split strategies can be applied. We have experimented with three of them. At first, we tried to use some kind of statistics. The central point for all P stationary points in the box was computed by a simple formula:

$$Center = \frac{1}{P}\sum_{i=1}^{P} X_i \tag{5}$$

Then a vector of relative deviations, or dispersions, was calculated for this central point:

$$Dispersion = \frac{1}{P}\sum_{i=1}^{P}(Center - X_i)^2 \tag{6}$$

Finally, the coordinate

$$k = arg\max_i Dispersion[i]$$

was chosen. The box was subdivided in two by plane, crossing the *Center* point and perpendicular to k-th coordinate axis. This strategy gave rather good results but it is also a little bit expensive from computational point of view. Thus another approach was

introduced. Instead of using all P stationary points contained in a box for calculations described above we suggested to consider only two of them with best function values. This strategy is used by default in current implementation. At last, we tried an additional rule which prevented splitting plane from intersecting with any stationary point in the box, but it required even more computations than in the first case, and gave no significant improvement as probability of such an intersection is very small.

4 How to use this program

In this section we illustrate how to write the complete program using our code. The short example program is given below for the purpose:

```
001 #include ”process.h”
002 extern const int DIM;
003 extern double F(TVector);
004 int main()
005 {
006   TBox Bounds(DIM);
007   Bounds.Right(0)=10.0;
008   Bounds.Left(0)=-10.0;
009   ...
010   long int Memory=640000;
011   long int Time=300;
012   char FileName[]=”results.out”;
013   double Accuracy=0.001;
014   double ARadius=0.1;
015   double FBand=0.001;
016   double DFStep=0.0001;
017   int Random=0;
018   int Start=DIM;
019   TProblemFFDD Prob(F,&Bounds,FBand,DFStep,FileName);
020   TMethod Met(Accuracy,ARadius,Random,Start);
021   TProcess Proc(&Prob,&Met,Memory,Time);
022   Proc.DoProcess();
023   return TRUE;
024 }
```

In line 001 the header file *process.h* which contains all necessary declarations is included. Lines 002 and 003 contain declarations of external objects: integer constant *DIM* - dimensionality of the problem, and real function F of TVector - function to be minimized. Of course, it is not necessary to use dimensionality constant, and here it is given just for illustrative purpose. Function can also be defined in the same file.

From line 004 C function **main** begins. In line 006 object *Bounds* of class TBox is created. It will contain function constraints, which are assigned further in lines 007 and 008. Here we only show initialisation of the first component, and ellipsis in line 009

represents the part of code where the rest of components are initialized. Naturally, user is free to choose how to perform initialisation of bounds: in linear part of code, in loop or in some other way.

In the part of code from line 010 to line 018 all the parameters needed to start search are defined. In this example we assign some values which are not essential and probably they will be changed in a real application. Two integer values - *Memory* and *Time* represent memory and time limits. Character string *FileName* contains a name of file where results should be written. Two real values - *Accuracy* and *ARadius* define the local search accuracy and radius of attraction respectively. Real values *FBand* and *DFStep* give band for minimal function values and step for calculating first derivatives. And, finally, integer values *Random* and *Start* represent number of random points and number of points to start local search. We should note that it is not necessary to define special variables for all these parameters but they can be written directly in the parameter lists of constructors.

In line 019 object *Prob* of class TProblemFFDD is created. This class is derived from class TProblem and supplies procedures for calculating function, given as a C function (see line 003), and for calculating first derivatives by using divided differences. Parameters of constructor are the following: function to minimize, pointer to bounds object, band for minimal function values, step for calculating first derivatives and results file name.

Object *Met* of type TMethod is created in line 020. Parameters of its constructor are the following: local search accuracy, radius of attraction, number of random points and number of points to start local search.

The last object needed to start running is created in line 021 - object *Proc* of class TProcess. The following parameters are passed to its constructor: pointer to problem object, pointer to method object, memory limit and time limit.

The whole search process is performed by call of a single function in line 022 - function DoProcess() of object Proc. It runs until the time limit is up. When the search is finished all the information is available through object Proc, namely, the lists of boxes can be read and modified, the solutions list and results file can be read and modified as well. After modifying some data process can be restarted. The results file will contain sequential blocks of data showing the progress in finding minimizers. Each block has the following format:

NUMBER OF F(X) AND F'(X) CALCULATIONS
    number of function and first derivative calculations on the moment
    when the modification of the results list occured. Modified results
    list is printed after this header.
RESULTS LIST
    results list in format:
        F(X) VALUE 1 : (X) VALUE 1
        F(X) VALUE 2 : (X) VALUE 2
        ...

Function **main** exits in line 023 and returns boolean TRUE as a result. As one can see, only about 30 lines of code are needed to write the complete program. We dare to assume

that our program is quite easy to use and it allows for an easy control of the search data. For deeper understanding please refer to the program source code and header files. These sources are available on request to the authors.

References

[1] Kaj Madsen and Serguei Zertchaninov (1998) *A New Branch-and-Bound Method for Global Optimization.* IMM-REP-1998-05, Department of Mathematical Modeling, Technical University of Denmark, DK-2800 Lyngby, Denmark.

[2] Kaj Madsen, Serguei Zertchaninov and Antanas Žilinskas (1998) *Global Optimization using Branch-and-Bound.* Submitted to *Global Optimization.*

[3] M.J.D. Powell (1970) *A Hybrid Method for Non-Linear Equations.* in "Numerical Methods for Nonlinear Algebraic Equations", P. Rabinowitz, ed. 87-114.
